# Transnational telepathology consultations using a basic digital microscope: experience in the Italy-Slovenjia INTERREG project “Patient without borders”

**DOI:** 10.1186/1746-1596-6-S1-S25

**Published:** 2011-03-30

**Authors:** Donatella Intersimone, Viviana Snoj, Franca Riosa, Nicola Bortolotti, Sandi Sverko, Carlo A Beltrami, Vincenzo Della Mea

**Affiliations:** 1 Section of Pathology, University Hospital of Udine, Italy; 2 Department of Pathology and Cytology, General Hospital Izola, Izola, Slovenia; 3 Medical Informatics, Telemedicine & eHealth Lab, Dept. of Mathematics and Computer Science, University of Udine, Italy

## Abstract

**Background:**

In recent years, a number of technological advancements started to modify the long standing appearance and functionalities of traditional optical microscopes used in Pathology and other medical fields. In fact, at present many new tools for microscopical visualization exist that are based on digital imaging, robotization, and remote communication. Such tools are typically adopted in activities ranging from education to telediagnosis to remote consultation.

Present paper describes the features of a basic digital microscope that has been tested to verify its performance for occasional remote consultation inside an international project between Italy and Slovenija, funded by Interreg initiative of the European Regional Development Fund.

**Methods:**

The system is composed by a pair of digital microscopes (Leica DMD108, Leitz Microsystems, Wetzlar, Germany) associated to a high resolution videoconferencing systems (Tandberg 990, Lysaker, Norway). The systems are connected through the Internet. Sixty histology and cytology cases have been collaboratively diagnosed between two Pathology Institutes to verify the diagnostic performance of the system, regarding the image quality point of view as well as time needed for diagnosis. The system has also been tested for compatibility with standard videoconferencing software.

**Results:**

No discrepancies between local and remote diagnoses have been identified, with diagnosis time reasonably close to typical microscope observation times. Time needed for most operations is not far from that needed on a traditional microscope, except for startup.

**Conclusions:**

The system can be considered usable as a standard microscope, and also for occasional remote consultations.

## Background

The traditional optical microscope is made for individual examination of samples through the eyepiece, and for this application it is very ergonomic and efficient [1]. However, microscopes are also used for discussing difficult cases as well as for teaching [2]. Both activities are inherently involving more than one observer, for which special microscopes are available with more than one eyepiece.

In the last years, the availability of digital imaging systems drove to novel ways of sharing images for the above purposes [3]. In fact, research microscopes almost always present a second light path made for attaching photographic cameras, which may also be used for digital cameras and video cameras. The latter are able to provide a real time source of digital images that can be directly displayed on a video monitor, thus allowing examination of images by more than one examiner. However, the image quality provided by video cameras can be inadequate for some applications, in particular at low magnifications, as it does not match the optical resolution of the microscope.

In addition to that, digital images also gave momentum to research fields like image analysis and telepathology [4].

For relatively long time since the availability of digital imaging systems, microscope manufacturers did not change the basic shape and functions of microscopes, concentrating instead on providing high quality photo and video cameras, viewing stations, and software. Other systems were directed to telepathology in its various meanings, i.e. for remotely exchanging images with some expert consultant, either in real time or store-and-forward way [5].

Only recently devices that could be defined as digital microscopes started to appear, aimed at routine examination of specimens [6]. The first commercial device of this kind was Nikon Coolscope, that encloses the equivalent of a traditional microscope in a computer-like case, without any eyepiece but with a direct output on a screen, and fully robotized control of microscope stage and objectives.

Another system of the same kind of more recent development is the digital microscope Leica DMD108 (Leitz Microsystems, Wetzlar, Germany). It is a basic digital microscope with manual interaction (versus the robotized one on the Nikon Coolscope), aimed at routine work. With an additional videoconferencing module (Tandberg), it can become a tool for occasional telepathology consultations or distant teaching sessions.

In the present paper we present a technological assessment exercise on the Leica DMD108 digital microscope, aimed at establishing whether the image quality provided is adequate for collaborative viewing of pathology images.

## Methods

### Systems

Assessment has been carried out between two workstations, one in the Institute of Pathology at the University of Udine, Italy and the other in the Institute of Pathology at the Hospital of Izola, Slovenia.

Each workstation was composed by a Leica DMD108 (Leitz Microsystems, Wetzlar, Germany) digital microscope connected by means of an hardware integration module to a high definition videoconferencing system (Tandberg 990, Lysaker, Norway). The videoconferencing system was in turn connected to the Internet.

### Assessment

Aims of our assessment regarding the overall system were:

- to understand whether it can be considered a substitute for a microscope,

- to evaluate diagnostic quality of images, and finally

- to verify the reliability of communications.

For the first aim, we examined the behaviour of the digital microscope by measuring time needed for main operations and features of the embedded software.

To test the system for collaborative discussion of cases, as well as to evaluate diagnostic quality a remote encounter programme was established, with week-based frequency, to examine 60 cases of interest for both institutes. We also evaluated optical and digital resolution of images on both static and dynamically delivered images.

During encounters, discussion time for each case was recorded as well as discussion outcome and errors and problems occurred during the communication session (to verify communication reliability).

Cases were mostly equally provided by the two institutes of Pathology involved in the study. Involved personnel was first briefly trained by three Authors of the present paper (NB, SS and VDM), during two encounters in the Izola and in the Udine Hospital.

## Results

### System features

The DMD108 digital microscope has manual stage and focus movements like in a traditional microscope, and robotized objective revolver. In both installations, objectives included the following magnifications: 5x, 10x, 20x, 40x, 63x.

Real time image acquisition and display occured at 1280x960 pixels, 24 bit depth. For the videoconference mode, images were scaled down to 1024x768, to allow real time transmission with reasonable bandwidths.

Apart from looking directly, it is possible to capture still images at a resolution of 3 MPixels (2048x1536). In this case, frame position on the slide was recorded with the image, in order to be able to return to the same place (although there is no motorised stage to do it automatically). Images can be stored on a USB drive, or on some file server on the network. When connected to a network, images can also be sent through email directly from the system interface.

**Figure 1:**
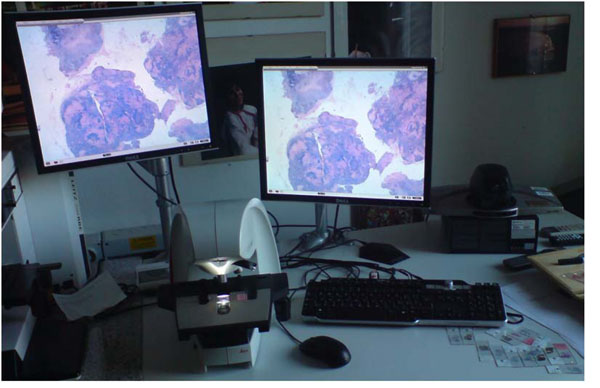
The system

Display is not an integral part of the microscope, so any monitor can be attached to the system. In our setup, two 19” 4:3 monitors were connected to the digital microscope, which show the same image when used locally, while in videoconference mode one is on the local microscope field, the other on the remote video. Also a keyboard and a mouse were attached to the microscope, to allow for input of textual descriptions. Figure 1 shows the setup in the Udine Institute.

**Figure 2:**
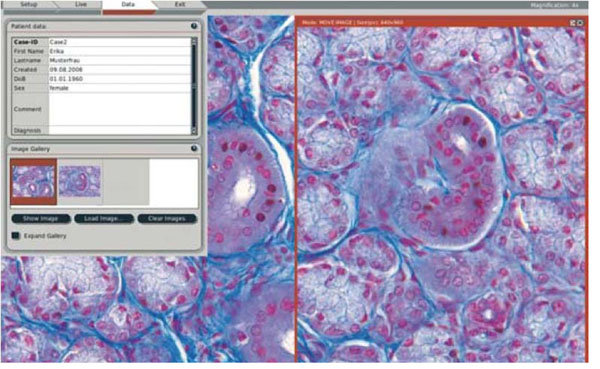
A sample screenshot from the interface

Another feature not traditionally available with microscopes is a macro overview of the slide, obtained by manually placing the slide in a different area of the stage.

Finally, it is also possible to directly trace lines, circles, comments on the image, as well as to measure lengths and areas, and also a voice recorder is available to record audio comments associated to the pictures.

Both systems were connected to the Internet, with specific ports open to allow for the videoconference. The Institute of Pathology at the University of Udine is hosted in an academic environment, and the public Internet, without closed ports, was already available. To avoid use of the hospital network, the hospital of Izola adopted a radio bridge to connect to an Internet provider.

An operating system provides a graphical user interface to the functions of the microscope. For example, different specimen stainings may be viewed in an optimized way by means of profiles that adapt colour curves to the specific staining to be displayed. It also provides some typical imaging services, like storage of still images on an USB drive or an external server, and delivery of images through email.

### System evaluation

We first evaluated system features regarding the quality of acquired and transmitted images. For this aim, we measured the size of fields acquired with the microscope.

Table 1 shows view field size, optical resolutions available with the used objectives, calculated by means of Rayleigh Law, and resolutions of respectively acquired and transmitted images.

**Table 1:** Optical, acquisition and videoconferencing resolutions for each objective

Objective magnification	Numerical aperture	Theoretical optical resolution (micron)	Pixel size of live video (micron)	Pixel size of transmitted images (micron)	Pixel size of still images (micron)
5x	0.12	2.29	2.04	2.55	1.28
10x	0.25	1.10	1.02	1.28	0.64
20x	0.40	0.69	0.51	0.64	0.32
40x	0.65	0.42	0.26	0.32	0.16
63x	0.75	0.37	0.16	0.20	0.10

As in most digital imaging applications in pathology, image resolution matches optical resolution only at higher magnifications, which however are the most frequently used to establish a final diagnosis.

Start-up time was about 1’05”. Shut-down time was 8 seconds. During every start-up it is necessary to calibrate the stage position by moving it in a corner. Time needed for any basic microscope operation (stage movement, focusing, objective change) did not differ from a traditional microscope. The computer interface for digital imaging operations was not based on commonly known operating systems, but was easy to use without previous knowledge.

Communications occurred flawlessly in all but one discussion session, which was not carried out because of lack of connection. An analysis of the situation recognized that the radio bridge used by the Izola Hospital to connect to Internet was suffering of problems due to bad weather.

### Collaborative discussion of cases

A total of 60 cases have been discussed between Udine and Izola, of which 30 cytologic samples, 27 histologic samples, 3 intraoperative samples. A pathologist and a biologist were present in Udine; a pathologist was present in Izola.

Discussion lasted 1’17” on average (minimum: 20”, maximum: 6’32”). Only among the very first cases, duration has been more than two minutes for some cases.

Outcome of discussion was an agreement on the diagnosis on 56 out of 60 cases. However, also the other discussions were concluded with an agreement: no disease in one case, need for further immunohistochemistry analyses in 2 cases, and insufficient experience on both sides for 2 other cases.

## Discussion

The tested digital microscope attempts at substituting a regular optical microscope, while providing also basic digital imaging facilities.

In our tests, the digital microscope behaviour has been very close to the one of a traditional microscope. Start-up time was of course higher than time needed to start a basic optical microscope, but not far from time needed to start a research microscope with robotized movements. Slightly more disturbing is the need for stage calibration at every start-up, but this can be avoided by leaving the system in standby mode.

Most common operations (focusing, stage movements, objective changes) were very similar in activation modality and execution time to the same operations carried out on a traditional microscope. According to pathologists, quality of images as seen on video is good at magnifications greater than 10x, and video refresh is sufficiently quick to see any change in focus and in captured field in real time. However, no quantification has been made to objectively evaluate these subjective assessments.

So, for the sake of pure microscopic examination, the present digital microscope can be considered a good substitute for a traditional microscope.

However, some concern exists regarding all digital imaging applied to microscopes, and compared, the ergonomics of the traditional microscope has two advantages:

• the view field is wider, due to the circular shape that cannot be digitally reproduced with an equivalent area; this may translate in more time needed for examination, like already shown for digital slides [8];

• the binocular vision helps in focusing attention on the slide, by making the external world invisible during examination.

While the former concern cannot be really overcome, regarding the latter it is probably matter of habits and attitudes, that will change when the “videogame generation”, more used to computer screens, will be in charge of microscope examinations [9].

On the other side, the system seemed easy to use, and only a short training period, during which part of discussion time was spent in understanding the device functioning, has been needed before reaching a good knowledge of the tool.

However, for the pathologist that only needs to look at slides alone, with only occasional need for digital images, there is no strict recommendation of leaving a traditional microscope for a digital one like this, because traditional functions are performed more or less in the same way.

The pathologist with need for collaboratively discussing cases (head of Institute, or teacher that routinely shows slides to residents) instead has different needs that can be easily fulfilled with the examined digital microscope. Current approaches to collaborative vision include the multi-headed microscope, which presents two or more eyepieces, or a video camera able to show images on a television-style monitor on the side of the microscope. The former represents the traditional way of teaching, but –except for the double eyepiece version- it is very cumbersome. The latter provides slightly sub-standard images, because resolution is often 600 lines according to television standards.

The digital functions provided by the microscope are to be compared with a traditional microscope enriched by a microscope photo camera. In this comparison, any digital microscope including the studied shows some clear advantages, including:

• integrated environment: no need for an external computer to be activated every time a picture is needed;

• easier focusing: not always aftermarket microscope cameras are installed in a perfect parafocality, so the sample cannot be focused directly at the eyepiece. Microscope cameras almost always allow for real time viewing on a computer or on an external monitor at a resolution much lower than the final one, thus making difficult to focus at the maximum level of detail.

The only microscope feature that was not functioning appropriately, at least in our device, was polarized light. It is an infrequently needed feature that however, when needed, cannot at present be reliably obtained with this microscope.

Whenever a pathologist needs to routinely acquire digital images, a digital microscope may become an efficient way of accomplish the task, in particular when coupled to an institutional server to store images.

Finally, we also preliminarily evaluated the digital microscope behaviour as a remote discussion tool. For this, the system was coupled with a videoconferencing system chosen by the microscope manufacturer and thus well integrated with it. To allow transmission through videoconferencing, image resolution has been reduced to 1024x768 pixels.

However, no specific problems have been recognised regarding image quality. Also ease of use of the combined microscope plus videoconference system has not been an obstacle to communications, so that after the first discussion sessions there has not been necessary to provide technical support.

As with other telepathology techniques [8], remote observation of cytologic specimens is more difficult due to the need of screening the whole slide (and thus with much interaction between two sites), while histologic and intraoperatory specimens may be observed first at very low magnification, and then only few interesting parts should be explored at high magnification.

The test was carried out in the framework of an international project between Italy and Slovenija, funded by Interreg Interreg initiative of the European Regional Development Fund. This allowed to experiment with a heterogeneous access to the Internet, that always but in one occasion behaved consistently. It should however be noted that implementing the system inside a protected hospital network may be not so easy due to the presence of firewalls and closed ports [10]. A close collaboration is needed with hospital ICT services to adequately configure the network.

Finally, occasional collaboration can be also done with remote users of videoconference systems, because the enclosed system is compliant with H.263 standard. Such systems could even be realised only by software, although the ones we tested were not able to negotiate the maximum resolution provided by the source videoconference system.

## Conclusions

The Leica DMD108 digital microscope has been assessed to verify its system functionality in routine telepathology work and in a collaborative case discussion scenario. The system can be considered usable as standard microscope, i.e. as a substitute for a traditional microscope, and also for occasional remote consultations.

## Competing interests

The authors declare that they have no competing interests.

## Authors' contributions

VDM and CAB designed the evaluation experiment; VDM wrote the first draft of the paper. NB and SS managed all technical details. DI, VS and FR did the remote collaborative diagnoses and discussed results. All authors read, revised and approved the article.
